# The Medial Ventrothalamic Circuitry: Cells Implicated in a Bimodal Network

**DOI:** 10.3389/fncir.2018.00009

**Published:** 2018-02-09

**Authors:** Tomas Vega-Zuniga, Dominik Trost, Katrin Schicker, Eva M. Bogner, Harald Luksch

**Affiliations:** Lehrstuhl für Zoologie, Technische Universität München, Freising-Weihenstephan, Germany

**Keywords:** microconnectomics, GLv, ICT, chicken, TSA, TDE, neutube, Vaa3d

## Abstract

Previous avian thalamic studies have shown that the medial ventral thalamus is composed of several nuclei located close to the lateral wall of the third ventricle. Although the general connectivity is known, detailed morphology and connectivity pattern in some regions are still elusive. Here, using the intracellular filling technique in the chicken, we focused on two neural structures, namely, the retinorecipient neuropil of the n. geniculatus lateralis pars ventralis (GLv), and the adjacent n. intercalatus thalami (ICT). We found that the GLv-ne cells showed two different neuronal types: projection cells and horizontal interneurons. The projection cells showed variable morphologies and dendritic arborizations with axons that targeted the n. lentiformis mesencephali (LM), griseum tectale (GT), ICT, n. principalis precommissuralis (PPC), and optic tectum (TeO). The horizontal cells showed a widespread mediolateral neural process throughout the retinorecipient GLv-ne. The ICT cells, on the other hand, had multipolar somata with wide dendritic fields that extended toward the lamina interna of the GLv, and a projection pattern that targeted the n. laminaris precommissuralis (LPC). Together, these results elucidate the rich complexity of the connectivity pattern so far described between the GLv, ICT, pretectum, and tectum. Interestingly, the implication of some of these neural structures in visuomotor and somatosensory roles strongly suggests that the GLv and ICT are part of a bimodal circuit that may be involved in the generation/modulation of saccades, gaze control, and space perception.

## Introduction

The ventral thalamus of birds is characterized by the presence of a group of nuclei located in close apposition. These neural structures include the griseum tectale (GT), n. lentiformi mesencephali (LM), n. geniculatus lateralis pars ventralis (GLv), and n. intercalatus thalami (ICT). They share an absence of projections to the telencephalon, and most of them send efferents to the diencephalon, mesencephalon, and metencephalon.

**Figure 1:**
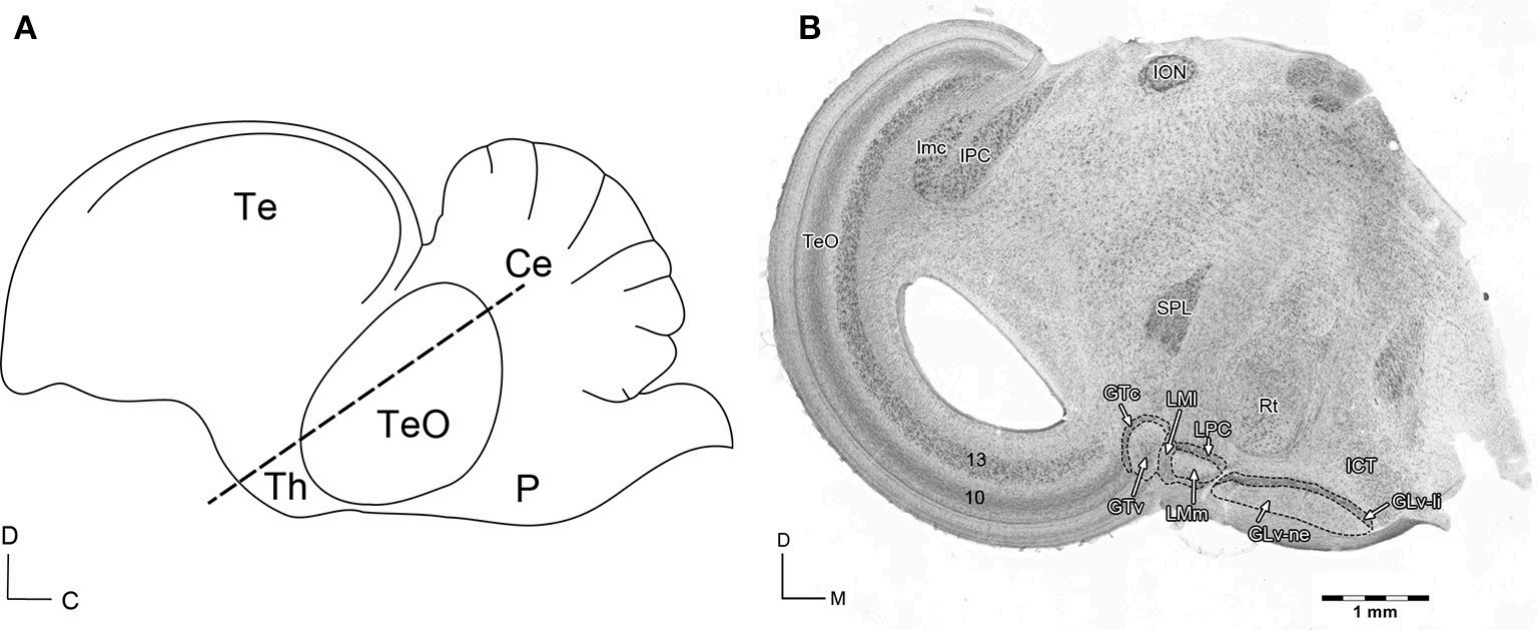
**(A)** Schematic view of the sagittal *Gallus gallus* brain. The dashed line indicates the site of the section in **(B)**. D, dorsal; C, caudal. **(B)** Giemsa staining of a 60 μm section that shows the diencephalic nuclei griseum tectale (GTv, GTc), n. lentiformis mesencephali pars lateralis (LMl) and pars medialis (LMm), n. laminaris precommissuralis (LPC), n. geniculatus lateralis pars ventralis-lamina interna (GLv-li) and -neuropil (GLv-ne), and n. intercalatus thalami (ICT). D, dorsal; M, medial. Modified with permission from Vega-Zuniga et al. (2016).

The GT is a pretectal retinorecipient structure that has two cell populations targeting the optic tectum, LM, GLv, and ICT (for details see Gamlin and Cohen, 1988; Vega-Zuniga et al., 2016). The adjacent structure LM is also a pretectal retinorecipient nucleus that has many different cell types projecting to specific targets in the dorsal thalamus, nBOR, inferior olive and cerebellum (for details see Freedman et al., 1975; Clarke, 1977; Zayats et al., 2003; Pakan et al., 2006; Iwaniuk et al., 2009; Wylie et al., 2009; Vega-Zuniga et al., 2016). Regarding the neurochemical profile, the GT and LM have a glutamatergic identity (Islam and Atoji, 2008; Atoji, 2011; Vega-Zuniga et al., 2016). Medial to the LM follows the Glv. This nucleus is a conspicuous and elongated ventrothalamic structure that (in the dorsoventral axis) lies between the n. rotundus and optic tract (see Figure 1). It is composed of two main layers: the lamina interna (GLv-li) and neuropil (GLv-ne) (Tombol et al., 2004; Vega-Zuniga et al., 2016). This latter part receives projections from the retina, optic tectum and visual Wulst (Karten et al., 1973; Crossland and Uchwat, 1979; Guiloff et al., 1987; Gamlin and Cohen, 1988; Tombol et al., 2004; Vega-Zuniga et al., 2014, 2016). The single cell microcircuitry of the GLv-li has been well characterized and contain neurons with complex dendritic arbors and projection patterns that span several diencephalic and mesencephalic nuclei such as the LM, n. laminaris precommissuralis (LPC), n. principalis precommissuralis (PPC), TeO, ICT, and an auto-terminal in the GLv-ne (Vega-Zuniga et al., 2016). The GLv-ne, on the other hand, has been described only morphologically, suggesting that some neurons have no projection to other structures while others might have (Guiloff et al., 1987; Guiloff, 1991; Tombol et al., 2004). Immunohistochemical and *in situ* experiments indicate that the GLv-li and GLv-ne possess a GABAergic identity (Sun et al., 2005; Vega-Zuniga et al., 2016). It is worth mentioning that although the function of the GLv is still unclear, it has been frequently implicated in optokinetic modulation (Gioanni et al., 1991) and head/eye orienting movements (Pateromichelakis, 1979; Guiloff et al., 1987; Guiloff, 1991; Harrington, 1997; Vega-Zuniga et al., 2016). Dorsomedial to the GLv, we find the ICT. This nucleus has a diffuse arrangement with no clear boundaries and layering. It is classically described as a non-retinorecipient structure. Nevertheless, intraocular tracing experiments in the chicken show sparse retinal endings in this area (BrainMaps, 2017). The ICT is innervated by the TeO, GT (Crossland and Uchwat, 1979; Gamlin and Cohen, 1988; Wild, 1989b; Vega-Zuniga et al., 2016) and visual suprachiasmatic nucleus (vSCN) (Cantwell and Cassone, 2006). In addition, the ICT also has inputs from the lumbar spinal cord via the spinothalamic pathway (Schneider and Necker, 1989) and from the dorsal column and external cuneate nuclei (DCN/CuE) (Wild, 1989a). The ICT projects to the GLd (Wild, 1989b) and TeO (Wylie et al., 2009). Also, Gamlin and Cohen (1988) suggested as unpublished observation that the ICT targets the nucleus pontis medialis (PM).

Previous experiments showed that the GLv-li has a significant collateral projection to the ICT (Vega-Zuniga et al., 2016). Hence, the single cell anatomy suggests a close synaptic interaction at the level of the ventral thalamus between the GLv-li and ICT. Nevertheless, it is unknown whether the GLv-ne also targets the ICT, and if the ICT projects back to either the GLv-li or GLv-ne. To unravel the entire medial ventrothalamic micro-circuitry, single cell morphology and micro-projection pattern of the GLv-ne and ICT cells are needed. In this study, we used a chicken slice preparation that conserves the GLv-ICT connectivity. We describe the detailed morphologies and micro-projection patterns of the cells located in the GLv-ne and ICT. We found that there is a highly complex organization of the GLv-ne and ICT neurons. Furthermore, they are connected with the pretectum and optic tectum. The unraveling of the medial ventrothalamic circuitry provides a solid single-cell anatomical basis to forthcoming experiments that aim to clarify the neuronal dynamics of this circuit implicated in visuomotor tasks. Also, it will allow direct comparisons with homologous neuronal populations in other vertebrates including mammals.

## Methods

### Animals

Forty chick hatchlings (*Gallus gallus*; P1–P3) were used in this study. Eggs were obtained from a local breeder (Hatchery Hoelzl, Moosburg, Germany) and incubated at 37°C and 70% humidity. *In vitro* procedures were approved by the Munich Veterinary Animal Care Committee and conformed to National Institute of Health guidelines on the ethical use of the animals. Efforts were made to minimize the number and suffering of the animals utilized in this study.

### Slice preparations

Chickens were anesthetized with a mixture (3:1) of Ketamine (50 mg/ml; Inresa Arzneimittel) and Rompun (2%; Bayer), at 37.5 and 5 mg/kg respectively. Subsequently, they were decapitated, and the skull was opened at the midsagittal line. The brain was carefully removed and transferred to ice-cooled (4°C) sucrose-substituted Krebs solution (210 mM sucrose, 3 mM KCl (Sigma, U.S.A.), 3 mM MgCl2·6H2O, 23 mM NaHCO3, 1.2 mM NaH2PO4·6H2O (Laborbedarf-Vertrieb GmbH, Germany), 11 mM D+-glucose). The telencephalon and cerebellum were cut through the connection with the rostral thalamus and cerebellar peduncles, respectively. Then, the brain was cut midsagittally. The two hemispheres were embedded in agar at 40°C and cooled down (1.5% Agar in HEPES solution: 290 mM sucrose, 3 mM KCl, 3 mM MgCl_2_, and 5 mM HEPES; Sigma Chemical Co., USA). Each tectal hemisphere was aligned in an oblique transverse plane (for details see Vega-Zuniga et al., 2014). Both parts were cut into 1,000 μm slices with a vibratome (VF 200 Compresstome, Precisionary Instruments Inc., USA). Slices were moved to ACSF solution (120 mM NaCl, 3 mM KCl, 1 mM MgCl_2_·6H_2_O, 23 mM NaHCO_3_, 1.2 mM NaH_2_PO_4_·1H_2_O, 11 mM D+-glucose) and kept in an interface chamber bubbled with Carbogen (95% oxygen, 5% CO2) for recovery at room temperature for 1 h. Then, the slices were slightly submerged within ACSF in the same chamber at room temperature.

### *In vitro* extracellular injections

**Figure 2:**
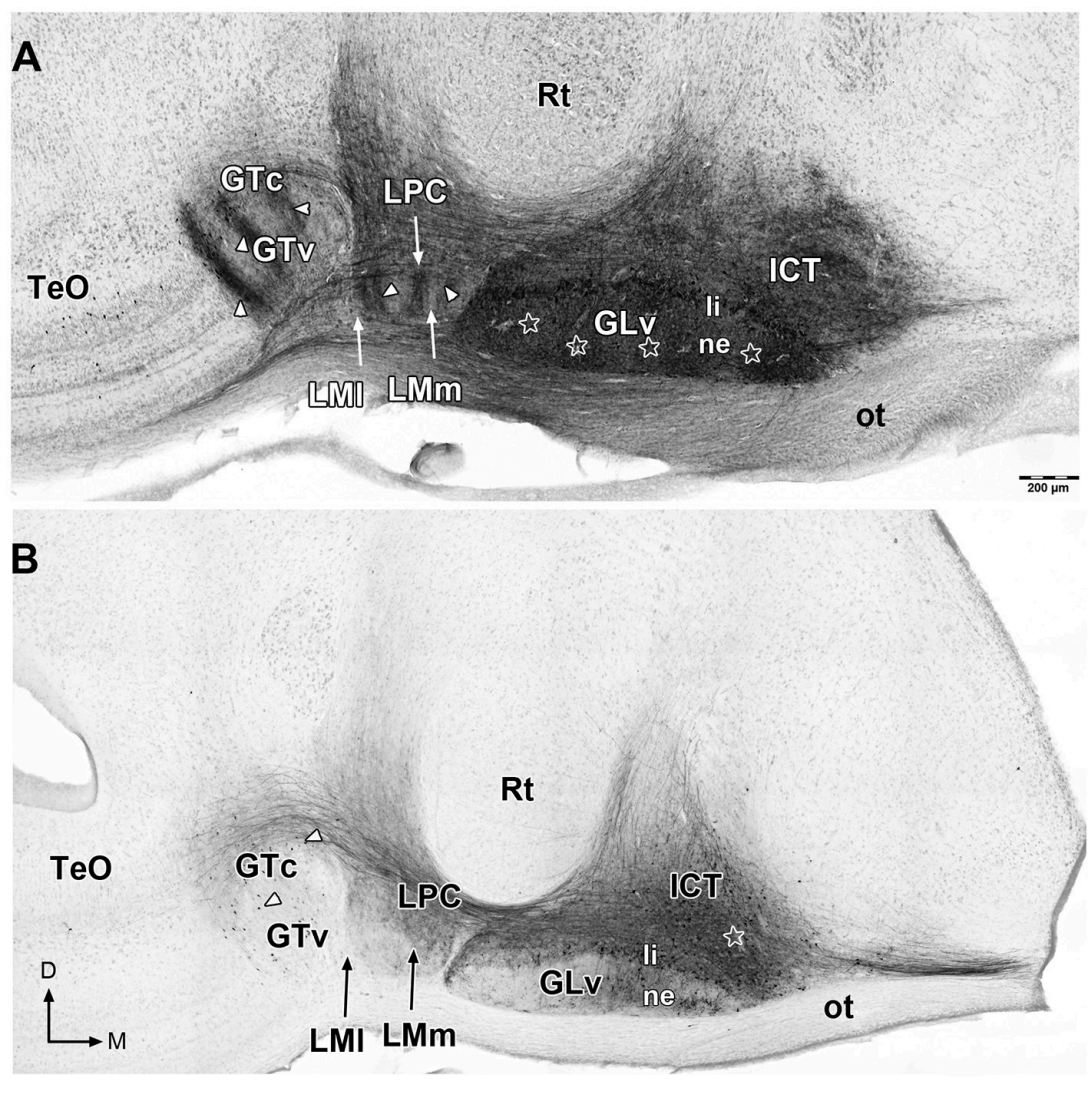
Giemsa counterstaining of 60 μm sections that shows retrograde and anterograde labeling of neurons and processes (white arrowheads) in the ventral thalamus and pretectum after *in vitro* injections in the GLv-ne **(A)** and ICT **(B)** (white stars). Note in **(A)** the clear topographic pattern in the LM and GT, and the massive non-topographic labeling in the ICT after GLv-ne injections. D, dorsal; M, medial. Orientation in **(B)** applies to **(A)**.

Borosilicate glass (GB100-8P, 0.58 × 1.00 × 80 mm; Science products GmbH, Germany) was used to produce pipettes with a tip diameter of 20 μm using a three-stage microelectrode puller (P-97, Sutter Instrument Co., U.S.A.). The glass-electrodes were filled with oil and incorporated to a Nanoliter 2000 injector (World Precision Instruments, U.S.A.). Electrodes were filled with Biocytin 8.5% (hydrochloride, Sigma-Aldrich, Germany) and dissolved in phosphate buffer (PB) 0.1 M (pH 7.4). The slices were submerged in a chamber with carbogenated ACSF solution. Under microscope control and with a microdrive (Maerzhaeuser, West-Germany), small injections of 13.8 nL tracer-solution throughout the GLv and ICT were done (Figure 2). Then, the slices were left 4 h in carbogenated ACSF solution. Slices with Biocytin injections were fixed overnight in 4% paraformaldehyde in 0.1 M phosphate buffer (PFA/PB). After this, they were moved to 30% sucrose (in PB) for 2 h, and sectioned to 60 μm with a sliding-microtome (Microme HM 400 E, GMI, USA). To view the staining, a heavy-metal-intensified DAB protocol was applied (for details see Vega-Zuniga et al., 2016). Briefly, to block endogenous peroxidase, sections were incubated in H_2_O_2_ solution [3% (wt/vol) H_2_O_2_] for 30 min. The tissue was then washed (8 × 10 min) in PB 0.1M at 4°C, and incubated in 0.1% (vol/vol) avidin-biotinylated HRP complex (ABC) solution containing 0.5% (vol/vol) Triton X-100 for 1 h. Afterwards, the tissue was moved into 0.026% diaminobenzidine/Ni-Co with 0.03% H_2_O_2_ for 6 min. Using gelatin subbed-slides, sections were mounted and counterstained with Giemsa (Sigma-Aldrich). Finally, they were dehydrated in an isopropanol series that ended in Xylol and coverslipped with DPX (Sigma-Aldrich, Germany).

### *In vitro* intracellular filling revealed with DAB

Borosilicate glass (GB150 F-8P, 0.86 × 1.50 × 80 mm, filament; Science products GmbH, Germany) was used to produce pipettes with a resistance of 30–60 MΩ using a microelectrode puller (Sutter Instrument P-97, USA). The pipettes were filled with 8.5% Biocytin (hydrochloride, Sigma-Aldrich, Germany) and 0.5% sulforhodamine 101 (Sigma-Aldrich) dissolved in 0.5 M KAc (pH 5.7). The tissue was incubated in acridine orange (10 mM, Sigma-Aldrich) for 20 min. The slices were moved to a slice chamber on a microscope stage that was continuously perfused with ACSF solution (RT 21/22 °C). The single slices were hold steady with a platinum wire. Recordings were done with an intracellular amplifier (WP Intra 767, World Precision Instruments, USA). Using a microscope (Nikon Y-FL, Eclipse E600FN, Japan), pipettes were moved through the brain-tissue with a micromanipulator (MWS-1A Narishige Co., Japan). After a cell was impaled, the tracer solution was injected with a positive current of 1.2 nA for 3 min. After a positive filling observed through rhodamine fluorescence, the tissue was left for 4 h in carbogenated ACSF solution. Subsequently, slices were fixed overnight in 4% paraformaldehyde (0.1 M phosphate buffer, PFA/PB), cryoprotected with 30% sucrose (in PB), and cut to 60 μm sections using a sliding microtome (Microme HM 400 E, GMI, USA). To visualize the filled neurons, a slightly adapted heavy-metal-intensified DAB protocol was applied (for details see Vega-Zuniga et al., 2016). Briefly, the tissue was incubated in H_2_O_2_ solution (3% (wt/vol) H_2_O_2_) for 30 min to block endogenous peroxidase. Then washed (8 × 10 min) in PB 0.1 M at 4°C, and moved to 0.5% (vol/vol) avidin-biotinylated HRP complex (ABC) solution containing 0.5% (vol/vol) Triton X-100 for 1 h. Next, samples were left in 0.026% diaminobenzidine Ni-Co for 10 min. The chromogenic reaction ocurred by adding H_2_O_2_ (final concentration 0.01%) for 60 s. Lastly, the tissue was mounted in gelatin-subbed slides, counterstained with Neutral Red (Sigma-Aldrich), dehydrated in an isopropanol-Xylol series, and coverslipped with DPX as mentioned above.

### *In vitro* intracellular filling revealed with fluorescence

Slices with biocytin-filled neurons were fixed overnight in PFA 4% and then washed three times in PB. Subsequently, slices were left in 0.5% (vol/vol) avidin-biotinylated HRP complex (ABC) solution that contained PBS (4% NaCl)-Tx 0.5% (vol/vol) for 24 h at 4°C. The slices were washed in PB, and then incubated with tyramide signal amplification (TSA) (for details see Krabichler et al., 2017). Briefly, sections were incubated in 0.0001% biotin-tyramide (IRIS Biotech GmbH, Marktredwitz, Germany; Cat# LS-3500, Lot. 1407008) and 0.003% H_2_O_2_ in 0.05 M borate buffer, pH 8.5, for 24 h at 4°C. Finally, and after washing in PB, slices were incubated in Streptavidin-Alexa 546 (Thermo Fisher Scientific, Waltham, MA, USA) diluted 1:500 in PBS (4% NaCl)-Tx 0.5% for 24 h at 4°C.

### Analysis

For optical analysis, slices were cleared with 2,2′-thiodiethanol (TDE; Sigma-Aldrich, St. Louis, USA) dissolved in PB 0.1 M (for details see Aoyagi et al., 2015). Slices were incubated in an ascending TDE concentration solution for 1 h at 42°C per treatment (20, 40, and 60%). Confocal microscopy was done using an Olympus Fluoview FV1000/BX61 (Olympus, Tokyo, Japan). Images were processed with Fiji (Schindelin et al., 2012; RRID:SCR_002285). For neuronal reconstruction, Neutube (Feng et al., 2015) was used. Then, .SWC files were analyzed and animated with Vaa3D (Peng et al., 2010) and Photoshop CS5.

## Results

### Extracellular injections into the ventral thalamus

To study the GLv-ICT connectivity, oblique-coronal slices containing the GLv and ICT were obtained (Figure 1). To make sure that the connection between these nuclei was included in the slices, small extracellular biocytin injections were performed in the GLv-ne and ICT. After a series of adjacent small injections in the GLv-ne (Figure 2A), a widespread staining pattern was observed throughout the tectum, pretectum, and ventral thalamus. These experiments showed a massive labeling of processes in the ICT with no clear topographic organization. In addition, labeling was observed in the LM, showing a precise topography of the neural processes (Figure 2A). Also, retrogradely stained neurons in the GTc were observed. These cells were closely located and regularly spaced with an obvious topography. The TeO also had labeled neurons located in layer 10b. Additionally, stained fibers were distributed throughout the pretectal PPC and deep layers of the TeO. Nevertheless, in these structures, no topography was observed (Figure 2A).

On the other hand, injections into the ICT (Figure 2B) showed a widespread labeling pattern, nevertheless, less intense than the one observed with the GLv-ne injections. Retrogradely labeled cells were found in the GLv-li, GLv-ne, GTc, and GTv, and in layer 14 of the TeO. Neural processes were observed in the GLv-li, GLv-ne, LPC, LMm, GTc, PPC and TeO (Figure 2B). The nucleus rotundus (Rt), which is adjacent to the ICT and GLv, was free of labeled cells or neural processes in both GLv-ne and ICT injections.

### Intracellular filling of the Glv-ne

To elucidate the morphology and microcircuitry of this structure, intracellular fillings in the GLv-ne were made *in vitro*. These experiments led to the identification of five cell types.

**Figure 3:**
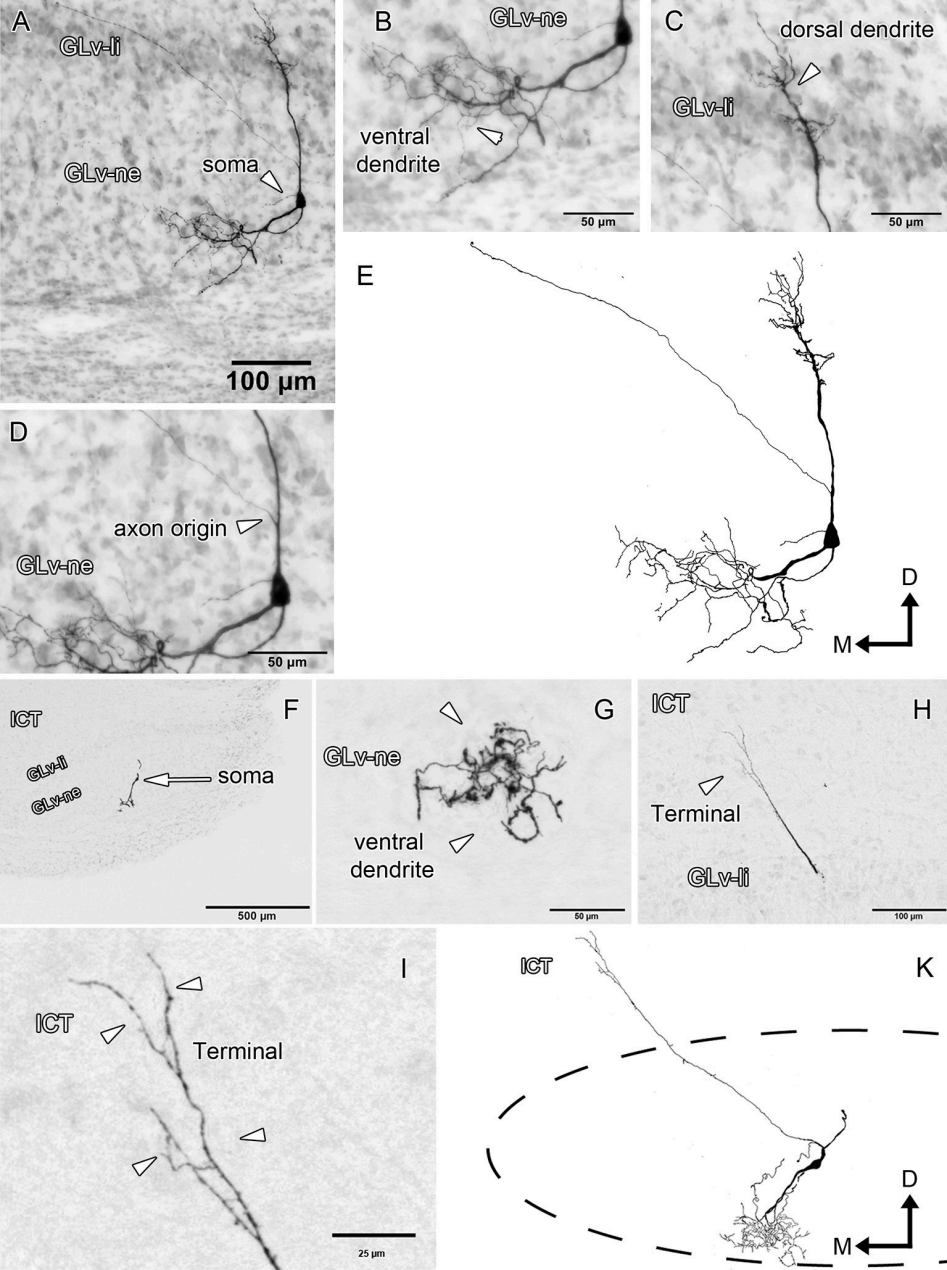
Intracellular filling of two representative GLv-ne type I cells. **(A–I)** Photomicrographs showing soma location, axon origin, dendritic processes and terminal ending. Arrowheads indicate the different neuronal components. **(E,K)** Reconstruction of the biocytin-filled neurons. D, dorsal; M, medial. Orientation in **(E,K)** applies to **(A–I)**.

Glv-ne-Type-I cells (three cases; Figure 3, Supplementary Video 1) have round-triangular somata with ventral dendritic processes that extend into the ventral area of the Glv-ne (Figures 3B,G), and a dorsal dendritic process that targets the GLv-li (Figure 3C). The axon originates from the dorsal dendritic trunk (Figures 3D,K) and projects mediodorsally, ending in the ICT where it ramifies in a small area (Figures 3H,I).

**Figure 4:**
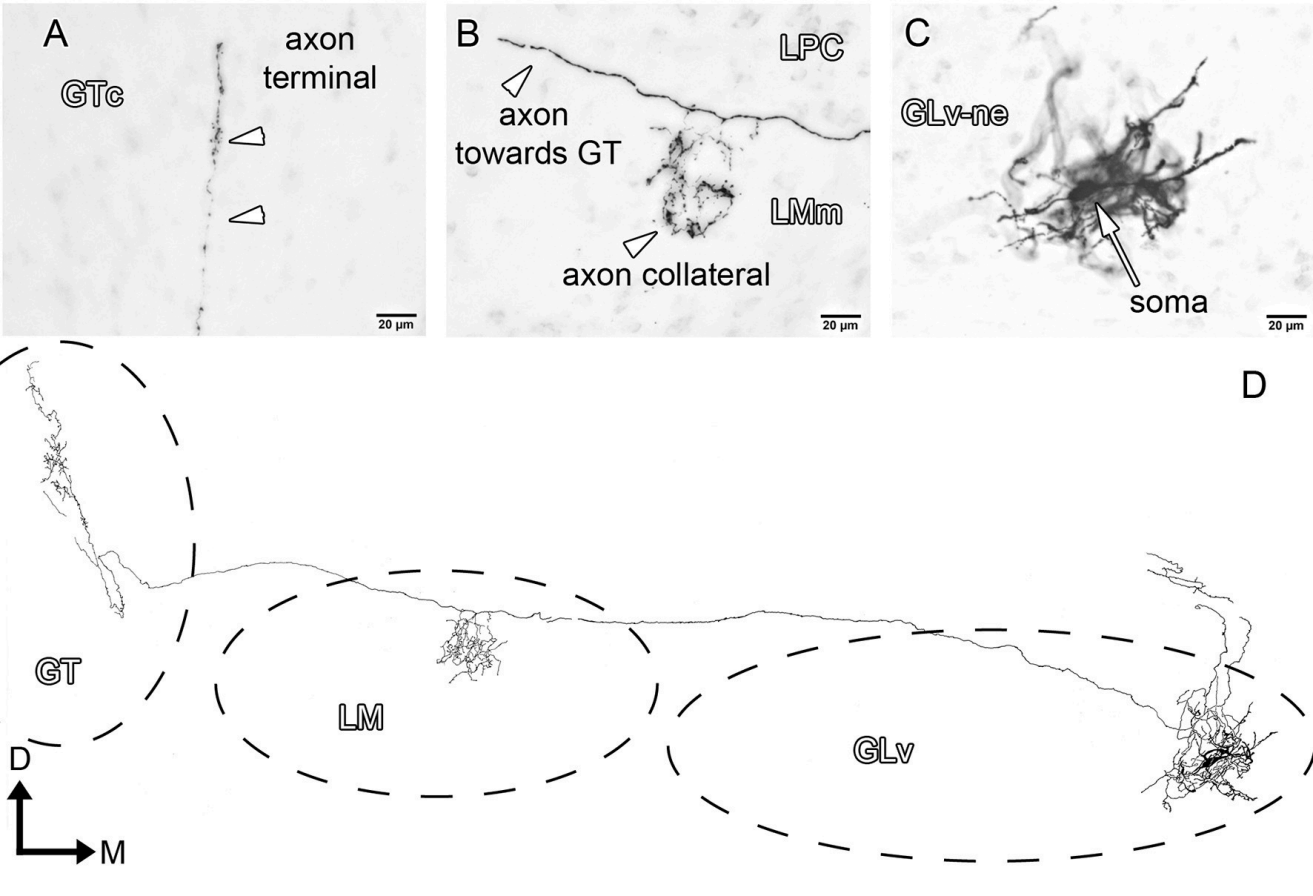
Intracellular filling of a representative GLv-ne type II cell. **(A–C)** Photomicrographs showing axon collaterals in LM/GT, and the location of the soma. White arrowheads indicate the different neuronal processes in LMm and GTc. **(D)** Reconstruction of the biocytin-filled neuron. D, dorsal; M, medial. Orientation in **(D)** applies to **(A–C)**.

GLv-ne-Type-II cells (three cases, Figure 4) show round somata with a dense multipolar dendritic tree that does not reach the GLv-li. The axon originates from the dorsolateral part of the dendritic tree, courses below the GLv-li and then enters the LMm from the medial part just below the LPC (Figure 4B). Here the axon leaves a restrict collateral terminal. The axon continues toward the GT where it enters through the ventral part, at the level of the medial GTv. The terminal extends dorso-ventrally until the ventral part of the GTc. It shows a very restricted mediolateral ending area that covers only a thin portion of the GT (Figure 4A).

**Figure 5:**
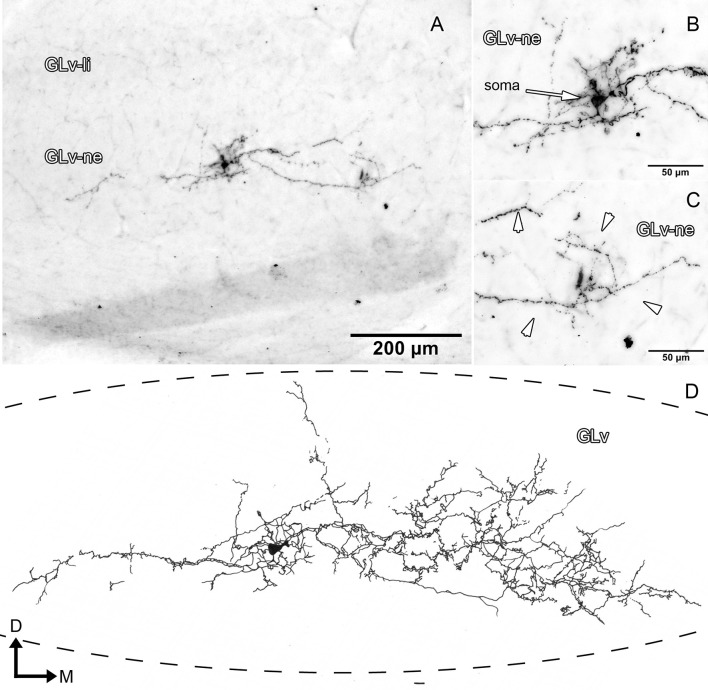
Intracellular filling of a representative GLv-ne type III cell. **(A–C)** Photomicrographs showing the neuronal extension, soma location, and dendritic processes (white arrowheads). **(D)** Reconstruction of the biocytin-filled neuron. D, dorsal; M, medial. Orientation in **(D)** applies to **(A–C)**.

GLv-ne-Type III cells (three cases, Figure 5, Supplementary Video 2) have round somata with a dendritic tree that extends mediolaterally through almost one-fourth of the whole GLv (Figures 5A,D). These cells did not have any recognizable axon within or leaving the GLv toward other neural structures.

**Figure 6:**
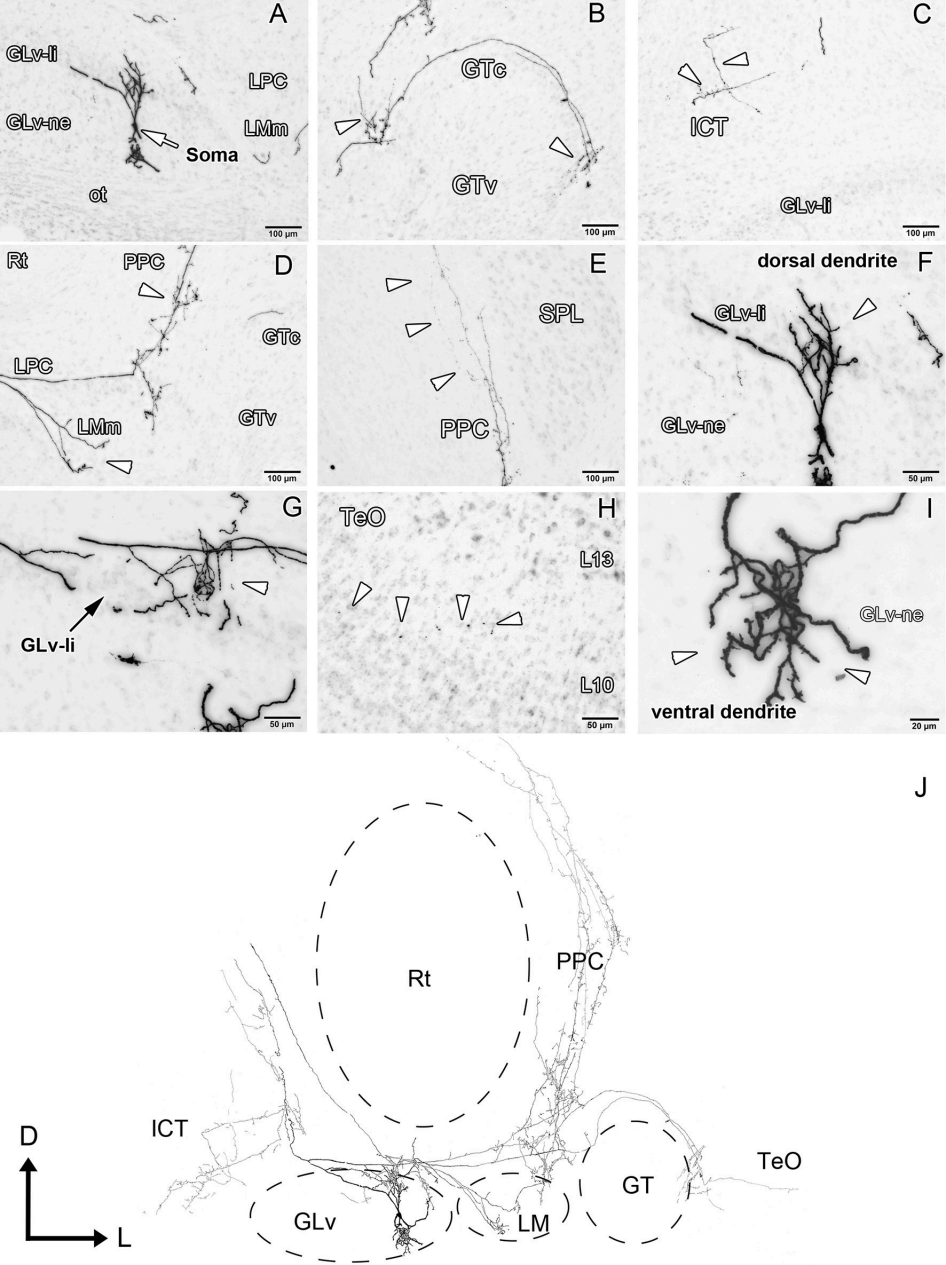
Intracellular filling of a representative GLv-ne type IV cell. **(A–I)** Photomicrographs showing the soma location and corresponding dendritic processes/axon collaterals throughout different nuclei. White arrowheads indicate the different neuronal processes. **(J)** Reconstruction of the biocytin-filled neuron. Note the impressive extent a single GLv-ne type IV cell has throughout the thalamus, pretectum and optic tectum. D, dorsal; L, lateral. Orientation in **(J)** applies to **(A–I)**.

GLv-ne-Type IV cells (three cases, Figure 6, Supplementary Videos 3, 4) show fusiform somata with a bipolar dendritic tree organization. The ventral dendrites extend into the ventral portion of the GLv-ne where they form a small restricted arborization field (Figure 6I, Supplementary Video 3). The dorsal dendritic tree extends into the GLv-li area (Figures 6A,F, Supplementary Videos 3, 4). The axon originates from the dorsal dendrite. In the region of the GLv-li, the axon splits into several axon collaterals, one of which ends in the area of the dorsal dendrite (Figure 6G). The next collateral projects laterally and splits into new terminals that end in the LMm (Figure 6D). The other axon collateral continues, generating new branches with endings in the LPC and LMl area (Figure 6D). Here, the axon divides again and generates two clear projections: the first one goes all the way through the GTc and leaves a collateral terminal in the lateral part of the GTc near the TeO (Figure 6B). Then, a very thin axon continues through the TeO, ending in layer 10 of the TeO (Figure 6H). The second axon, which splits at the level of the LPC, courses dorsally, leaves terminals through all the PPC area (Figures 6D,E) and terminates in the dorsal thalamus, presumably in the DLL area (Figure 6J). The medial portion of the axon projects dorsally where it leaves a thin wide-spread collateral that ends in the ICT area (Figure 6C, Supplementary Video 4). The axon that continues dorsal presumably targets the pontine nuclei.

**Figure 7:**
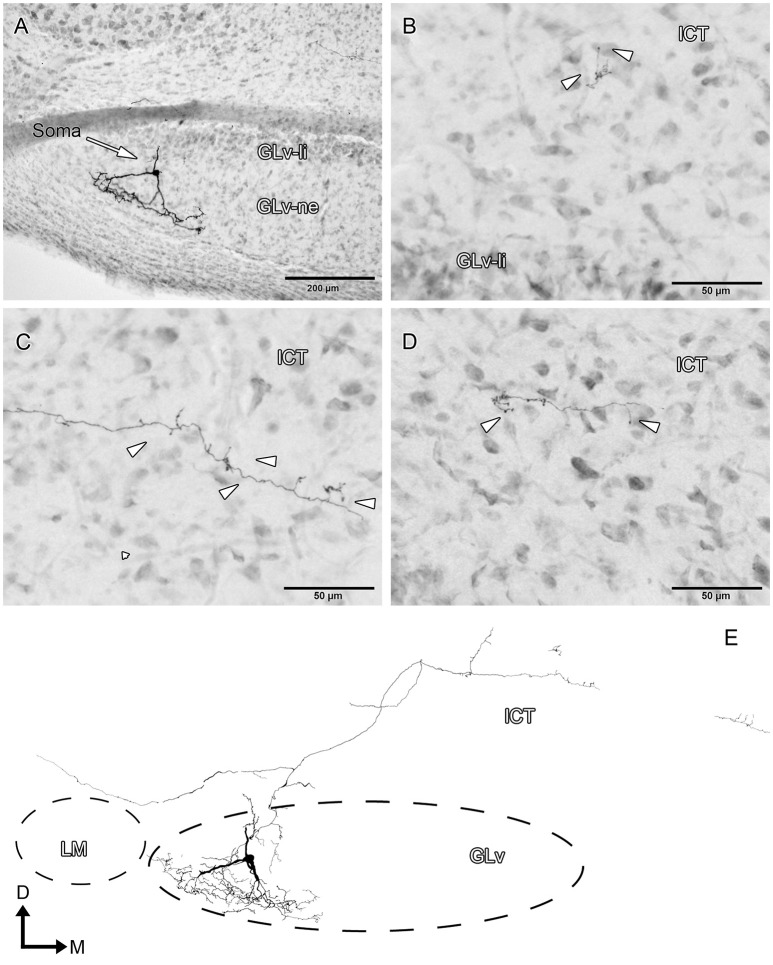
Intracellular filling of a representative GLv-ne type V cell. **(A–D)** Photomicrographs showing the soma location, dendritic processes, medial axon and terminal ending in ICT. Arrowheads indicate the different neuronal processes. **(E)** Reconstruction of the biocytin-filled neurons. D, dorsal; M, medial. Orientation in **(E)** applies to **(A–D)**.

GLv-ne-Type V cells (two cases, Figure 7, Supplementary Video 5) have triangular somata with two conspicuous dendritic trees that extend ventrally separated by approximately 90° (Figures 7A,E, Supplementary Video 5). In addition, a dorsal dendrite extends up to the GLv-li. From this latter dendrite emerges a medial axon that ends in the ICT (Figures 7B–E). A second collateral of the axon runs toward the LM without a definite ending (Figure 7E).

### Intracellular filling of the ICT

To clarify the microconnectomics of this structure, intracellular fillings in the ICT were performed *in vitro*. These experiments lead to the identification of one cell population.

**Figure 8:**
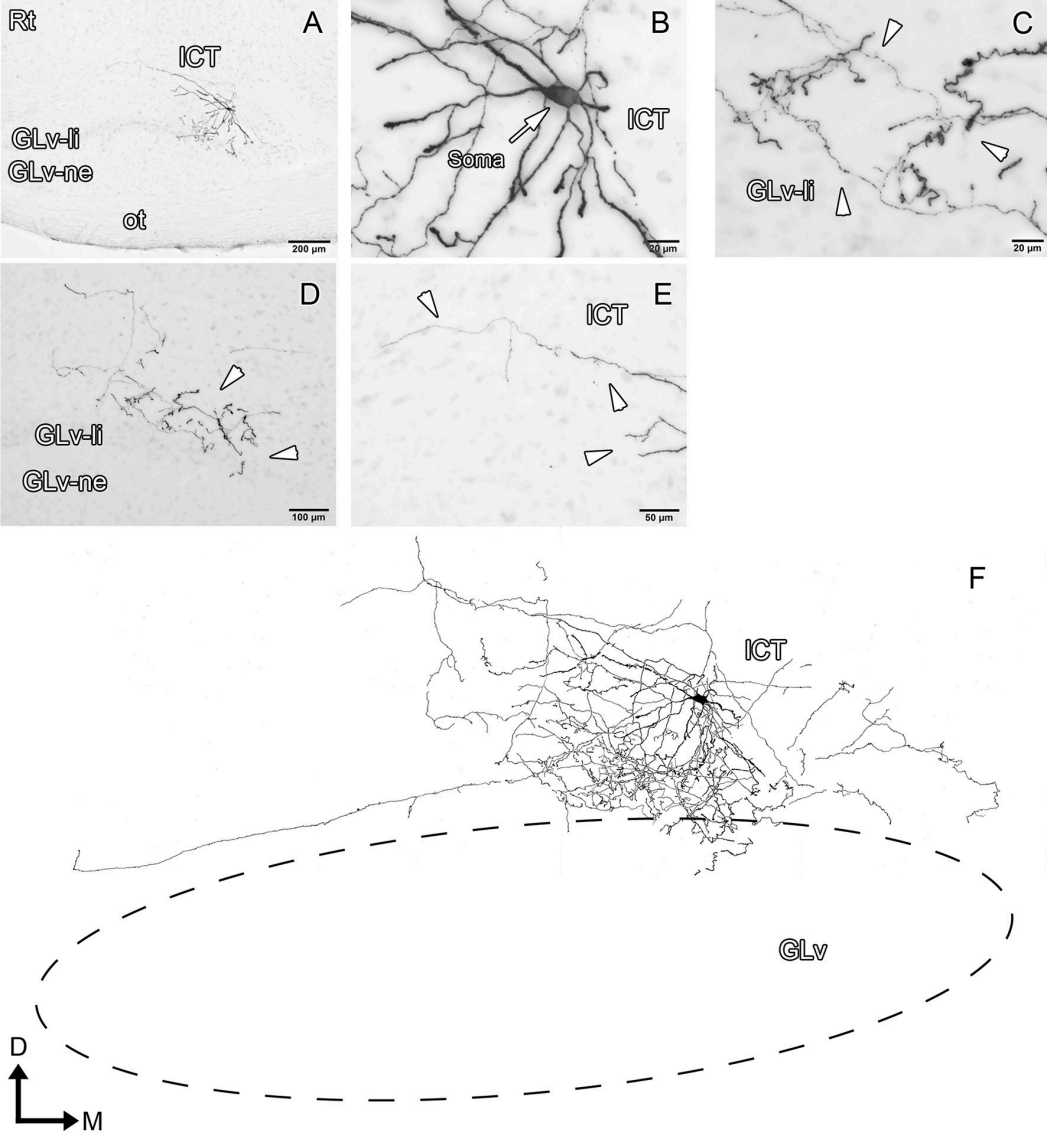
Intracellular filling of an ICT cell (case1). **(A–E)** Photomicrographs showing the neuronal extension, soma location, and neural processes (white arrowheads). **(F)** Reconstruction of the biocytin-filled neuron. Note that the dendritic processes become denser in the GLv-li region. D, dorsal; M, medial. Orientation in **(F)** applies to **(A–E)**.

**Figure 9:**
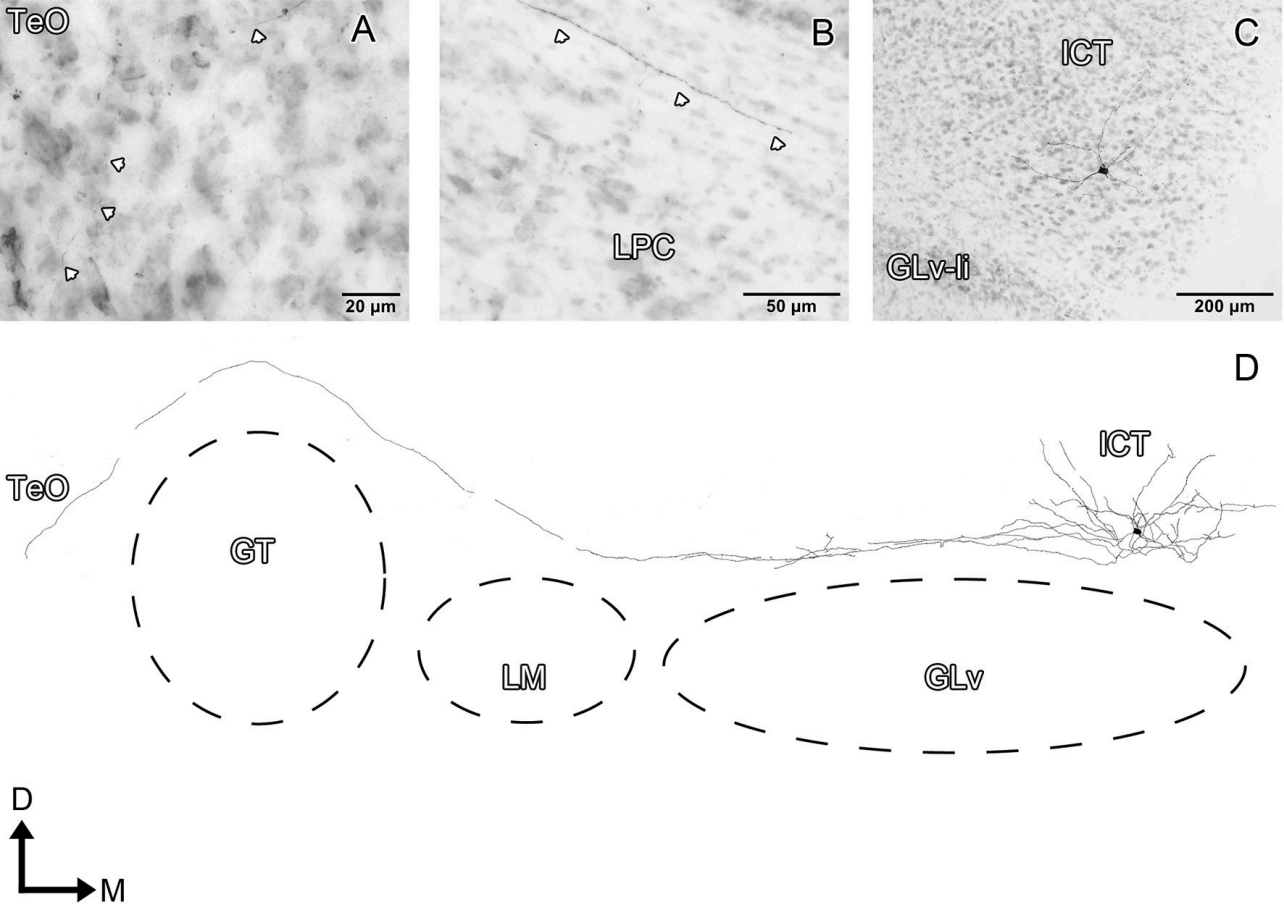
Intracellular filling of an ICT cell (case 2). **(A–C)** Photomicrographs showing the axonal entrance to the TeO, axon path, and soma loaction (white arrowheads). **(D)** Reconstruction of the biocytin-filled neuron. D, dorsal; M, medial. Orientation in **(D)** applies to **(A–C)**.

**Figure 10:**
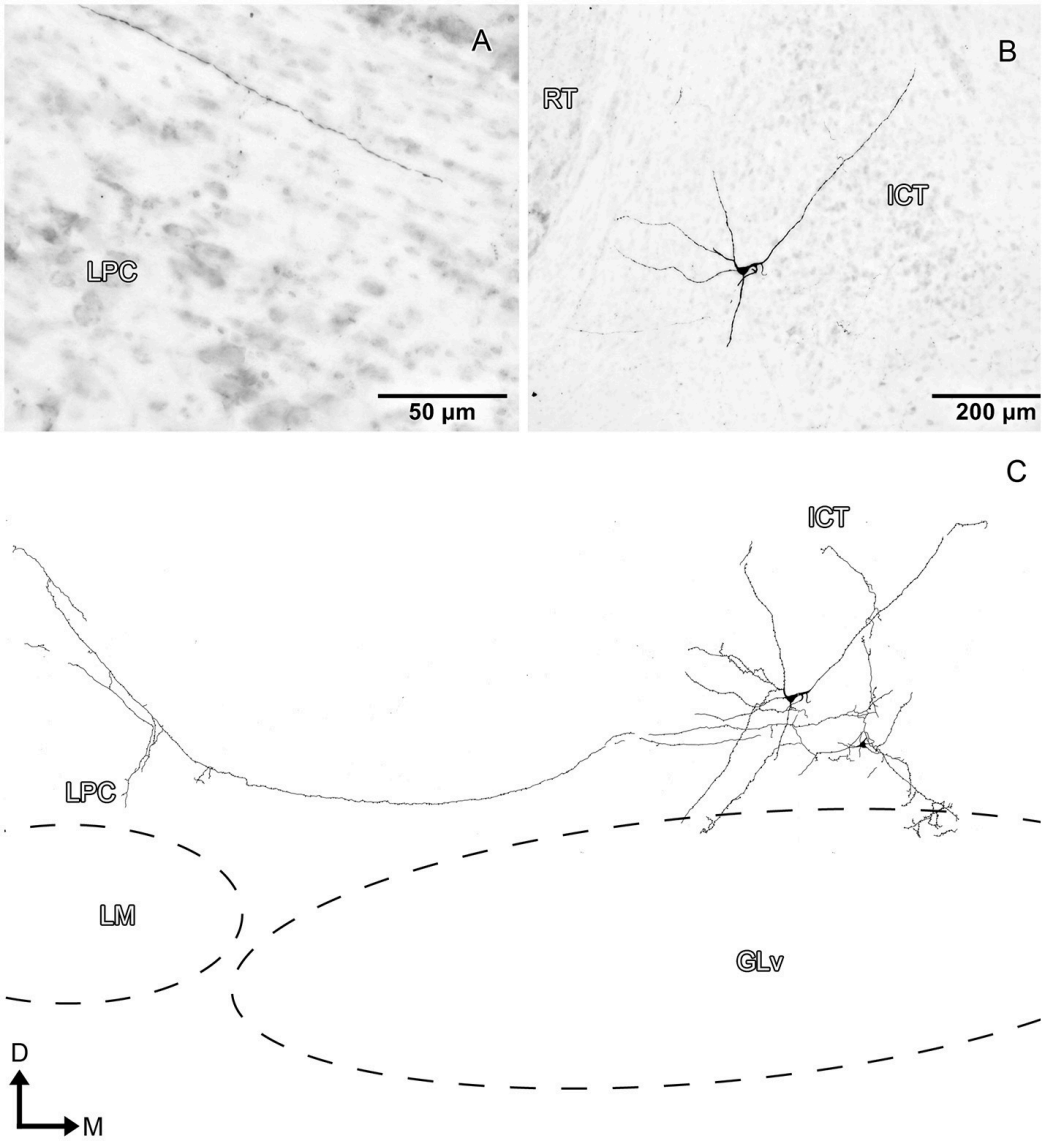
Intracellular filling of an ICT cell (case 3). **(A,B)** Photomicrographs showing the axon collateral over LPC and the location of the soma (white arrowheads). **(C)** Reconstruction of the biocytin-filled neuron. Note that the dendritic processes reach the GLv-li region. D, dorsal; M, medial. Orientation in **(C)** applies to **(A,B)**.

ICT cells (five cases) have oval somata with a multipolar dendritic tree organization (Figures 8–10, Supplementary Video 6) that has a widespread distribution within the nucleus. In case 1 (Figure 8), the dendrites have a massive and dense distribution throughout the ICT that reaches, in the ventral portion, the lamina interna of the GLv (Figures 8C,D,F). The axon originates from a ventral dendrite and extends laterally just above the GLv-li. In case 2 (Figure 9), the dendritic tree spreads widely throughout the ICT but does not reach the GLv-li zone (Figure 9D). The axon originates from a ventrolateral dendrite and runs above the GLv, LM/LPC, and GT to finally enter the TeO between layer 10 and 13 (Figure 9A) where it presumably ends. In case 3 (Figure 10), cells show an extensive dendritic tree with dendritic spines (Figures 10B,C) that extends ventrally up to the GLv-li (Figure 10C). The axon originates from a ventral dendrite and runs laterally. At the level of the LM, the axon leaves a collateral in the LPC (Figure 10C).

## Discussion

**Figure 11:**
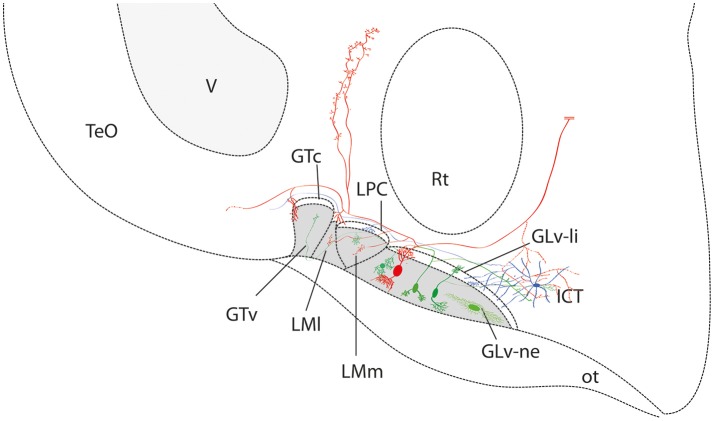
Summary diagram of the ventrothalamic neural circuitry. The parallel lines (in red) at the end of the axon shows that it continues beyond the confines of the slice. The retino-recipient region is represented with gray shading. Note that the GLv-li, LPC, and GTc are absent of retinal endings.

In this study, we describe the microconnectomics of cells located in the GLv-ne and ICT in the chicken. Our data show that these structures are strongly connected, particularly by neurons with large dendrites and axons that cover numerous nuclei. Cells in the GLv-ne have axons that produce terminals in the LPC, LM, GT, PPC, TeO, and ICT. Neurons in the ICT are also projection neurons with collaterals into the LPC and TeO (Figure 11).

### Morphology and projection pattern of the GLv-ne cells

Classical Golgi studies have identified five different cell populations in the GLv, i.e., GLv-li and GLv-ne (Guiloff et al., 1987; Guiloff, 1991; Tombol et al., 2004). In previous intracellular work, we described the morphology of neurons located in the GLv-li and found that the GLv-li is composed of mainly one neuronal population. This cell type showed complex dendrites and axons that extended through numerous diencephalic and mesencephalic structures (for details see Vega-Zuniga et al., 2016). In the present study, we characterized five cell types in the GLv-ne based primarily on the projection pattern. Therefore, a one to one comparison with previous studies is rather problematic since they were based only on morphology/soma size and not connectivity. For example, our type I, type II and type V may correspond to the “projection neurons (pn)” in Tombol et al. (2004) and type-I, type-II or type-IV in the Guiloff et al. study (1987). Nevertheless, the horizontal cells that we describe (type-III) are entirely consistent with the horizontal cells described by Tombol et al. (2004). The type-IV cells that we characterize are not present in any of the previously published studies. Furthermore, these cells are a remarkable novelty in this circuit. In respect to the shape, form and projection pattern they resemble neurons located in the GLv-li, that is, neurons with complex dendritic arborization and projection pattern that extend through several structures located in the ventral thalamus, pretectum, dorsal thalamus and optic tectum. Interestingly, apart from the location of the soma, the main difference between them is the target of the auto-terminal: GLv-ne type-IV auto-terminal ends in the GLv-li. On the contrary, GLv-li cells' auto-terminal mainly targets the ventral portion of the GLv-ne.

It is worth mentioning that the neuronal diversity that we found in the GLv-ne is very intriguing, specifically the way they connect to other structures. For example, type-I and type-II cells project to different targets, namely, the ICT and LMm/GT, respectively, whereas type-IV cells not only proceed to the same targets mentioned above, but to other structures such as the GLv-li, TeO, PPC, and dorsal thalamus. These different projection patterns may reflect different sub-functional pathways. Thus, suggesting that the GLv-ne is a neural structure where many different synaptic operations are taking place.

### Discrimination between the ICT and VLT

In the second part of this study we focused our interest in the ICT, a ventromedial diencephalic structure that has a close anatomical relationship with the GLv. We were able to characterize the ICT neurons present in our slice, covering a significant portion of the ICT. Nevertheless, the ICT is a diffuse structure (Gamlin and Cohen, 1988; Schulte et al., 2006) that merges in the rostral axis with another diencephalic nucleus known as the n.ventrolateralis thalami (VLT) (Karten and Hodos, 1967; Puelles et al., 2007). Remarkably, besides both being diffuse structures, they share considerable hodological similarities. The ICT and VLT receive afferents from the TeO, vSCN, DCN/CuE, and retina (sparse projections), whereas the efferent projections target the TeO. Moreover, after a thorough examination of the visual studies that mention the VLT and/or ICT, the question arises if these two allegedly separate structures are indeed the same nucleus. For example, in the anatomical study of Hunt and Künzle (1976) the region characterized as the VLT may be indeed part of the ICT. Furthermore, in Schulte et al. (2006), where they examined visual responses and afferent connections of the VLT, some recorded regions may correspond to the ICT. It is interesting to note that most authors do not mention the ICT and VLT at the same time. Rather, they speak about either VLT or ICT. This may happen, as mentioned before, because both structures have no clear boundaries, thus making it very difficult to discern where one ends, and the other begins. Hence, it seems to be that in vision literature the ICT/VLT topic has to be revised. Further experiments will be needed to clarify if they are indeed two separate structures or correspond to the same neural complex. For this study, we describe the cells of the ICT or more caudal part of the VLT/ICT complex.

### Morphology and projection pattern of the ICT cells

To our knowledge, there is no morphological characterization of ICT neurons in birds. Regarding the projection pattern, we confirm for the first time that ICT neurons extend only into the GLv-li and not to the GLv-ne. Our results suggest that these neural processes correspond to dendrites. Previous studies have shown an ICT projection to the vSCN (Cantwell and Cassone, 2006). In our preparation, we cannot confirm such a projection as this connection is severed in the slice.

In the pretectum, the ICT cells only target the LPC area, suggesting a specific connection with the LPC cells that project to the cerebellum (Pakan et al., 2006). Literature shows that ICT also target the TeO (Hunt and Künzle, 1976; Wylie et al., 2009). Although we have cases where the axon enters the TeO, the laminar localization of the axonal terminals could not be determined. Therefore, there is no clarity regarding which layer(s) of the TeO are connected with the ICT.

### Synaptic interactions in the medial ventrothalamic network

The GLv is innervated by topographic retinal projections that end in the GLv-ne region. Thus, cells located in the GLv-ne (projection- and interneurons) may be targeted either at the soma and/or dendrites by retinal ganglion cells with glutamatergic synapses (Guo et al., 1998). On the other hand, GLv-li cells are in contact with retinal inputs only at the level of their dendrites since the location of the somata is devoid of retinal terminals (Crossland and Uchwat, 1979; Vega-Zuniga et al., 2014). In all these GLv cells, AMPA receptors GluR4, GluR2/3, and GluR1 are expressed (Pires and Britto, 1997). This selective targeting enables specific synaptic contacts on the first neurons of the ventrothalamic circuitry. This way, compared to the GLv-li cells, the GLv-ne cells have potentially more contact-areas that may result in differential synaptic dynamics. In addition, retinal terminals in the GLv possess nicotinic receptors (Guo et al., 1998, 2005), which may be modulated by tectal vine-neurons located in layer 10b (Vega-Zuniga et al., 2014). Interestingly, it has been shown that cholinergic modulation in the GLv can result in excitatory as well as inhibitory responses through nicotinic and muscarinic receptors (Guo and Chiappinelli, 2002; Guo et al., 2010). Also, GABAergic synapses are expected to occur due to the GABAergic neurochemical profile of GLv cells (Veenman and Reiner, 1994; Vega-Zuniga et al., 2016), and the presence of interneurons and projection neurons that have terminals either in the lamina interna (GLv-li) or neuropil (GLv-ne). Hence, the GLv is a structure where glutamatergic, cholinergic and GABAergic synapses are taking place.

Regarding the GLv-ICT interaction, this process may happen at different synaptic levels. On the one hand, the cells that have a widespread terminal in the ICT may be either making contacts along the long and spiny ICT dendrites or contacting a larger number of ICT cells. On the other hand, the cells with a narrow terminal field may target the soma of ICT neurons or a particular portion of the dendritic field. Regarding the ventral dendrites that reach the GLv-li, we predict that this dendritic portion may receive synaptic contact from GLv-ne Type-IV neurons (which have collaterals in this particular area, see Figure 6G).

Although there are some studies (including this one) that addressed the problem of the ventro-thalamic connectivity, further physiological studies are needed to understand the synaptic dynamics between these two thalamic structures.

### Functional considerations of the GLv-ICT circuitry

In the past 30 years, the role of the GLv in vision has been a matter of debate with no clear conclusions. It has been suggested that it is involved in circadian rhythm (Harrington, 1997), chromatic discrimination (Maturana and Varela, 1982), optokinetic reflex (Gioanni et al., 1991), and visuomotor responses (Pateromichelakis, 1979; Guiloff, 1991; Harrington, 1997; Vega-Zuniga et al., 2016). Our series of microconnectomic papers (Vega-Zuniga et al., 2014, 2016) and the present study strongly suggests that the GLv is more related to optokinetic reflex and visuomotor responses than chromatic discrimination and circadian rhythms. In addition, in our previous paper we observed a strong projection from the GLv to the ICT that we further investigated in this study. The GLv-ICT connection is very striking since the ICT has direct afferents from the rhombencephalic nucleus DCN/CuE. The same projection also targets the n. dorsalis intermedius ventralis anterior (DIVA), which is the primary thalamic structure that has a connection with the somatosensory Wulst, i.e., the hyperstriatum accessorium (HA). Furthermore, electrophysiological experiments confirmed somatosensory receptive fields in DIVA neurons (Schneider and Necker, 1996).

Hence, the ICT is a nucleus that has, on the one hand, an ascending collateral projection from a demonstrated somatosensory pathway, and on the other hand, a massive collateral input from the retinorecipient GLv (that includes both laminae). These data strongly suggest that the ICT is a bimodal thalamic structure where two different sensory pathways interact. Interestingly, a similar bimodal/multimodal case occurs in the n. dorsolateralis posterior thalami (DLP), a dorso-thalamic structure that has afferents from visual, auditory and somatosensory pathways (Korzeniewska and Güntürkün, 1990). Note that this latter pathway, as mentioned above, is the one that leaves a collateral terminal in the ICT.

Thus, the results suggest that different sensory pathways interact at early stages of neural operations, generating a close dynamic coordination between each other. Consequently, we suggest that the GLv-ICT connection forms a key functional thalamic node where visual and somatosensory pathways interact in a precise and concerted manner. Furthermore, they may be part of a broader circuit that enables complex behaviors such as gaze control and space perception. Further physiological and behavioral experiments will be necessary to confirm or disprove this hypothesis.

## Summary

We used the intracellular filling technique to describe the morphology and projection pattern of neurons located in the GLv-ne and ICT. The results demonstrate an intricate connectivity pattern between a visual (GLv-ne) and a putative somatosensory (ICT) structure. This complexity is orchestrated primarily by various neuronal populations present in the GLv-ne. Furthermore, the cells located in the GLv-ne and ICT extend throughout several neural structures that include the pretectum and TeO, hereby connecting with the central visual pathways involved in vision. We suggest that this complex microconnectomics may be the structural basis that enables, at a thalamic level, the generation/modulation of saccades, gaze control, and space perception.

## Author contributions

All authors had full access to all data in this study and take responsibility for the integrity of the data and accuracy of the analysis. Study concept and design: TV-Z. Acquisition: TV-Z, DT, KS, EB. Analysis and interpretation: TV-Z, DT, KS, EB. Wrote the manuscript: TV-Z. Significant comments to the manuscript text: HL. Administrative, and material support: HL.

### Conflict of interest statement

The authors declare that the research was conducted in the absence of any commercial or financial relationships that could be construed as a potential conflict of interest.

## Supplementary material

Supplementary Video 1 Animated 3D reconstruction of a biocytin-filled GLv-ne type I cell.

Supplementary Video 2 Animated 3D reconstruction of a biocytin-filled GLv-ne type III cell.

Supplementary Video 3 Animated 3D reconstruction of a biocytin-filled GLv-ne type IV cell.

Supplementary Video 4 Animated 3D reconstruction of a biocytin-filled GLv-ne type IV cell.

Supplementary Video 5 Animated 3D reconstruction of a biocytin-filled GLv-ne type V cell.

Supplementary Video 6 Animated 3D reconstruction of a biocytin-filled ICT cell.
